# Antibodies Against the *Plasmodium vivax* Apical Membrane Antigen 1 From the Belem Strain Share Common Epitopes Among Other Worldwide Variants

**DOI:** 10.3389/fcimb.2021.616230

**Published:** 2021-03-16

**Authors:** Ana Caroline Barbosa França, Kátia Sanches Françoso, Rodolfo Ferreira Marques, Gustavo H. G. Trossini, Renan A. Gomes, Marinete M. Póvoa, Maristela G. Cunha, Eduardo L. V. Silveira, Irene S. Soares

**Affiliations:** ^1^ Department of Clinical and Toxicological Analyses, School of Pharmaceutical Sciences, University of São Paulo, São Paulo, Brazil; ^2^ Department of Pharmacy, School of Pharmaceutical Sciences, University of São Paulo, São Paulo, Brazil; ^3^ Instituto Evandro Chagas, Ananindeua, Brazil; ^4^ Instituto de Ciências Biológicas, Universidade Federal do Pará, Belém, Brazil

**Keywords:** malaria, *Plasmodium vivax*, apical membrane antigen 1, polymorphism, vaccine

## Abstract

Malaria is a human parasitic disease distributed in many tropical countries and caused by various *Plasmodium* species. *Plasmodium vivax* has the largest geographical distribution of the *Plasmodium* species and is predominant in the Americas, including Brazil. Only a small number of *P. vivax* vaccine formulations have successfully reached clinical trials relative to their *P. falciparum* counterparts. One of the candidate antigens for a blood-stage *P. vivax* vaccine is apical membrane antigen 1 (PvAMA-1). Due to the worldwide distribution of *Plasmodium* parasites, a high degree of variability has been detected in this antigen sequence, representing a considerable challenge to the development of a universal vaccine against malaria. In this study, we evaluated how PvAMA-1 polymorphisms influence vaccine-derived immune responses in *P. vivax* malaria. To this end, we expressed 9 recombinant protein representatives of different PvAMA-1 allelic variants in the yeast *Pichia pastoris*: Belem, Chesson I, Sal-1, Indonesia XIX, SK0814, TC103, PNG_05_ESP, PNG_62_MU, and PNG_68_MAS. After protein expression and purification, we evaluated the breadth of the immune responses derived from malaria-exposed individuals from the Amazon region. From 611 serum samples of malaria-exposed individuals, 53.68% of them reacted against the PvAMA-1 Belem through ELISA. Positive samples were further tested against recombinant proteins representing the other PvAMA-1 allelic variants. Whereas Sal-1, Chesson I and SK0814 variants were highly recognized by tested serum samples, Indonesia XIX, TC103, PNG_05_ESP, PNG_62_MU, and PNG_68_MAS were only slightly recognized. Moreover, polyclonal sera derived from C57BL/6 mice immunized with the PvAMA-1 Belem protein predominantly recognized Belem, Sal-1, Chesson I, SK0814, and Indonesia XIX through ELISA. Last, ELISA-based competition assays demonstrated that a previous interaction between anti-Belem polyclonal serum and Sal-1, Chesson I, SK0814, or Indonesia XIX proteins could further inhibit antibody binding to the Belem variant. Our human and mouse data suggest the presence of common epitopes or cross-reactivity between Belem, Sal-1, Chesson I, and SK0814 variants. Although the PvAMA-1 Belem variant induces strain-transcendent antibodies, PvAMA-1 variants from Thailand and Papua New Guinea may need to be included in a universal vaccine formulation to achieve protection against *P. vivax* malaria.

## Introduction

According to the World Malaria Report (WORLD HEALTH ORGANIZATION, 2019), an estimated 228 million people contracted malaria in 2018, resulting in 405 thousand deaths globally. *Plasmodium falciparum* and *Plasmodium vivax* are the most prevalent human malarial species worldwide, and *P. vivax* is the predominant malarial parasite in Asia and the Americas. Although *P. vivax* malaria is considered a benign infection because it exhibits lower parasitemia and milder symptoms than *P. falciparum* malaria, *P. vivax* infection is a highly debilitating disease. As hundreds of millions of cases of infection are annually related to *P. vivax*, the ensuing illness has a very important economic status. Given the difficult measures taken to control *P. vivax* malaria through conventional epidemiological methods and the increasing levels of parasite resistance to chemotherapy, the development of a protective vaccine against malaria has been considered a key priority (Draper et al., 2018; Antonelli et al., 2020).

The pre-erythrocytic *P. falciparum* candidate RTS,S is the first malaria vaccine to reach phase III testing. Currently, this vaccine has been in pilot implementation in three African countries (Rabinovich et al., 2017; Duffy and Patrick Gorres, 2020). Due to the complex life cycle of *Plasmodium* species, it has been suggested that a vaccine formulation only targeting the pre-erythrocytic stage of infection may not offer sterile protection against the disease. Moreover, *P. vivax* has the notable property of developing a dormant liver-stage, known as the hypnozoite, that is responsible for relapsed infections, making blood-stage vaccines particularly important for this condition (Tham et al., 2017). Thus, a multistage vaccine targeting antigens whose expression is derived from 2 or more phases of infection might be necessary to achieve sterile protection and to prevent symptomatology and disease worsening (Joyner et al., 2016).

Several blood-stage antigens have been identified as potential vaccine candidates (López et al., 2017; Tham et al., 2017) and apical membrane antigen-1 (AMA-1) is one of the most promising blood-stage antigen to compose a vaccine against apicomplexa parasites. *Plasmodium* AMA-1 is a type 1 transmembrane protein comprised by a pro-sequence, a cysteine-rich ectodomain, a transmembrane domain, and a C-terminal cytoplasmic domain (Nair et al., 2002). This antigen is expressed in the micronemes and apical surface of mature merozoites (Remarque et al., 2008) and belongs to the moving junction complex system that mediates the parasite internalization within the host cell (Remarque et al., 2008; Bargieri et al., 2013).

The tridimensional structure of the Sal-1 strain of the *P. vivax* AMA-1 protein was elucidated using a recombinant protein representing the entire protein ectodomain (Pizarro et al., 2005). The resolution of this structure confirmed the type of conformation initially suggested by the protein primary structure, consisting of three domains with 16 cysteine residues that form 8 disulfide bonds. Subsequently, the crystal structures of AMA-1 from other *Plasmodium* species (*P. falciparum* (Bai et al., 2005) and *P. knowlesi* (Vulliez-Le Normand et al., 2015)) were determined. The interaction between AMA-1 and the Rhoptry Neck Protein (RON) complex has been shown to be an important step for the host invasion by *P. falciparum* and *T. gondii* (Lamarque et al., 2011; Srinivasan et al., 2011). More specifically, AMA-1 interacts directly with the component RON2 of the complex, which is also identified in *P. vivax* (Bermúdez et al., 2018; Salgado-Mejias et al., 2019).

Several studies characterizing the naturally acquired human immune responses to the PvAMA-1 ectodomain were performed in malaria endemic areas from Brazil (Rodrigues et al., 2005; Morais et al., 2006; Múfalo et al., 2008; Vicentin et al., 2014; Sánchez-Arcila et al., 2015; Pires et al., 2018; Soares et al., 2020), Peru (Rosas-Aguirre et al., 2015), Sri Lanka (Wickramarachchi et al., 2006), India (Kale et al., 2019), Ethiopia (Keffale et al., 2019; Assefa et al., 2020), Indonesia (Surendra et al., 2019), Iran (Salavatifar et al., 2015) and other regions around the world (Kim et al., 2003; Cook et al., 2010; Xia et al., 2015). These studies confirmed the high immunogenicity of this protein during infections, especially in regions with higher levels of disease transmission and in individuals with recent infections. Furthermore, immunization with recombinant PvAMA-1 protein based on a Brazilian parasitic isolate elicited invasion-inhibitory antibodies against various Asian isolates of *P. vivax* (Vicentin et al., 2014).

Indeed, it has been reported that the PfAMA-1 genes are highly polymorphic (Thomas et al., 1990). However, vaccine formulations based on AMA-1 ectodomain sequences demonstrated protective immunity in animal models of human malaria (Remarque et al., 2008). Thus, this leading vaccine candidate has reached clinical trials (Dicko et al., 2008; Malkin et al., 2008; Thera et al., 2008; Ellis et al., 2009; Spring et al., 2009; Thera et al., 2010; Thera et al., 2016; Laurens et al., 2017; Sirima et al., 2017). The analysis of the nucleotide PvAMA-1 sequences derived from different parasite strains indicated a degree of polymorphism. Thus, the influence of this polymorphism must be further investigated in terms of its antigenicity (Arnott et al., 2013; Mueller et al., 2013). A recent Brazilian study evaluated the diversity of PvAMA-1 in various isolates and identified 19 haplotypes. Among these sequences, 33 nonsynonymous PvAMA1 amino acid (aa) sites were identified, and 20 of them were determined to be located in predicted B-cell epitopes (Bittencourt et al., 2020). The presence of gene polymorphisms in the sequence of a specific target antigen may compromise its usage as a vaccine candidate. Moreover, the protein folding may be affected, displaying a distinct conformation in comparison to the native counterpart, as well as epitopes within the polymorphic areas may lose immunogenicity (Angeletti et al., 2017).

Considering this gap in knowledge concerning the potential application of PvAMA-1 in a malaria vaccine formulation, we investigated 9 PvAMA-1 protein variants representing the global diversity of this antigen. More specifically, we investigated whether the immunity elicited against one PvAMA-1 variant (from the Belem strain) would cross-react with 8 other variants and thus indicate the presence of common epitopes among them. For this study, the antibody responses raised against the different variants in both human sera from malaria-exposed individuals from the Amazon region and murine sera from C57BL/6 mice immunized with PvAMA-1 Belem were evaluated by ELISA.

## Materials and Methods

### Sera Sample Collection

Human sera were collected from 611 residents of different malaria endemic areas in the state of Pará (Brazil) between 2006 and 2010. These samples were tested for malaria through microscopic parasitemia examinations, following the recommendations of the Brazilian Ministry of Health (http://bvsms.saude.gov.br/bvs/publicacoes/malaria_diag_manual_final.pdf), according to which results are given for 100 analyzed fields of the thick blood smear and parasitemia was expressed as the number of parasites per µl of blood; and 78 individuals were positive for *P. vivax* infection. The median age of the study participants was 31 years (ranging from 2 to 82 years), and they were 50.9% male and 49.1% female. Sera of unexposed individuals from southeastern Brazil were employed as negative controls (n=20). Ethics approval was provided by the Ethics Committee of the Evandro Chagas Institute (020/2006 and 0031/2010) and the School of Pharmaceutical Sciences, University of São Paulo (CAAE n°. 3.198.871/2019). Written informed consent was obtained from all participants or their guardians.

### Origins of PvAMA1 Alleles

**Figure 1:**
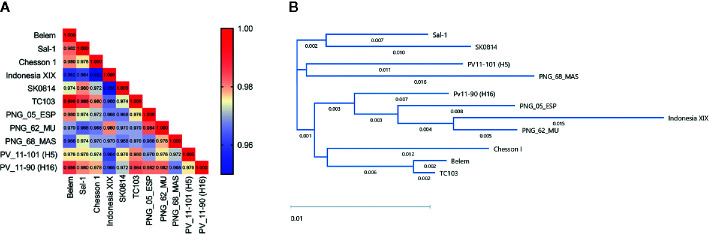
PvAMA-1 protein diversity analysis. The diversity of selected PvAMA-1 sequences from diverse geographical locations was compared among themselves. **(A)** Heatmap represents the identity scores among the different isolates ranging from 0.95 (blue) to 1.00 (red). **(B)** Dendogram of the phylogenetic tree obtained from full-length amino acid sequences of PvAMA-1 isolates using the BIONJ algorithm.

Nine alleles of the PvAMA-1 antigen (Belem, Sal-1, Chesson I, Indonesia XIX, SK0814, TC103, PNG_05_ESP, PNG_62_MU and PNG_68_MAS) were chosen from a previously published study [49]. The origins of these alleles, as well as their GenBank accession numbers, are listed in **Table 1**. After aligning the 9 allele sequences, 31 polymorphic aa positions were identified in the PvAMA1 ectodomain: 18, 6, and 7 polymorphic position were observed in Domains I, II, and III, respectively (**Figure 1**). The number of aa differences between any two PvAMA-1 variants ranged from 2 to 17.

**Table 1:** Worldwide distribution of the 9 PvAMA-1 variants.

PvAMA-1 variant	Country of origin	GenBank access code
Belem	Brazil	EU395595
Sal 1	El Salvador	XM_001615397
Chesson I	Malaysia	EU395596.1
Indonesia XIX	Indonesia	EU395598
SK0814	South Korea	GU476488
TC103	Thailand	FJ784917.1
PNG_05_ESP	Papua New Guinea	KC702448.1
PNG_62_MU	Papua New Guinea	KC702493.1
PNG_68_MAS	Papua New Guinea	KC702496

### Phylogenetic Sequence Analysis

Alignments of aa sequences and their relative identity scores were obtained through the ClustalW method. The phylogenetic tree with distinct PvAMA-1 protein sequences was generated with the BIONJ algorithm (Gascuel, 1997) using the DNASTAR Lasergene 17. Sequence identity matrix scores were generated using Bioedit Software version 7.2.

### Structural Modeling

Based on the comparative modeling strategy, tertiary protein structure models were constructed using the iTasser server (Yang et al., 2014). Each model was sequentially assessed for stereochemical quality using the WHATCHECK and QMEAN tools (Hooft et al., 1996; Benkert et al., 2011). Additionally, the sequences were submitted to the DIsEMBL server for protein disorder prediction (Abraham et al., 2015). Finally, the tridimensional protein structures were minimized using the AMBER99SB-ILDN force field in GROMACS 5.1.4 software. To this end, enough water molecules (TIP3P model), Na+ and Cl- ions were added as counterions to neutralize the system until physiological concentrations could be reached.

### Generation of Recombinant PvAMA-1 Proteins

The DNA sequences encoding aa 43 to 487 from the Belem, Sal-1, Chesson I, Indonesia XIX, SK0814, TC103, PNG_05_ESP, PNG_62_MU and PNG_68_MAS PvAMA1 alleles were codon-optimized for expression in *Pichia pastoris* yeast (Genscript). Three potential N-glycosylation sites were changed to prevent unwanted glycosylation (178N→S, 226N→D, and 441N→Q), as previously described (Vicentin et al., 2014). Additionally, all recombinant proteins contained a hexa-histidine tag at the C-terminus end, which enabled further purification through nickel resin affinity chromatography. DNA sequences were inserted into the pPIC9K vector (Invitrogen, Life Technologies Corporation USA, Inc.) using *Eco*RI and *Not*I restriction sites. The recombinant plasmids were linearized with *Sal*I (New England Biolabs Inc.) to transform the *Pichia pastoris* GS115 strain (his4^–^) (Invitrogen, Life Technologies Corporation USA Inc.) through electroporation. Transformed yeasts were subsequently grown on histidine-deficient media for the selection of His+ clones. In keeping with the instructions provided by the manufacturer of the Pichia Expression Kit (Invitrogen), clone selection with multiple inserts was performed on YPD (yeast extract – peptone – dextrose) plates containing geneticin (Sigma-Aldrich) (from 0.5 to 6.0 mg/mL).

Protein expression and purification were performed as previously described (Vicentin et al., 2014). Briefly, the selected clones were grown for 24 hours at 28-30°C with constant shaking (230 rpm) in BMGY medium (1% yeast extract (Sigma-Aldrich), 2% peptone (Sigma-Aldrich) and yeast nitrogen base without amino acids. Next, cells were harvested by centrifugation, solubilized in BMMY medium (BMGY with glycerol replaced by 0.5% methanol (Merck)) and cultured at 28-30°C with constant shaking at 230 rpm. The induction of protein expression was maintained by the daily addition of 1% methanol throughout the 96-hour incubation period. Cells were harvested by centrifugation, and the supernatant was filtered out using 0.45-µm membranes (Merck Millipore).

Recombinant PvAMA-1 proteins were purified through a two-step procedure (affinity and ion exchange chromatography). The yeast culture supernatants containing the solubilized protein were initially subjected to affinity chromatography using a HisTrap™FF nickel (GE Healthcare USA Inc.) column coupled to the FPLC ÄKTA Prime Plus system (GE Healthcare USA Inc.). The protein elution occurred against an imidazole gradient (15–400 mM, USB, Affymetrix USA Inc.) in phosphate–chloride buffer (20 mM NaH_2_PO_4_ (Labsynth Ltda.), 20 mM Na_2_HPO_4_ (Labsynth Ltda.), and 0.5 M NaCl (Labsynth Ltda.), pH = 6.0). Fractions containing the recombinant proteins were dialyzed and subjected to a purification step through ion exchange chromatography using the HiTrap™ QFF Column (GE Healthcare USA Inc.), which was also coupled to the ÄKTA system. Purified recombinant proteins were then dialyzed against phosphate-buffered saline (PBS) (8 mM NaH_2_PO_4_, 2.3 mM Na_2_HPO_4_, 130 mM NaCl (Sigma-Aldrich), pH = 7.4) overnight, with constant stirring at 4°C.

Protein concentrations were determined through densitometry analysis using ImageQuant™ TL version 8.1 software (GE Healthcare USA Inc.) and compared to calibration curves with defined concentrations of bovine serum albumin (BSA) (Invitrogen, Life Technologies Corporation USA Inc.).

### SDS-PAGE and Immunoblot Assays

The supernatants of *P. pastoris* cultures expressing the recombinant proteins were subjected to electrophoresis using 12% SDS-PAGE and stained with Coomassie Blue solution (Sigma-Aldrich) or electrotransferred onto Hybond N nitrocellulose membranes (GE Healthcare USA Inc.) for immunoblot analysis. Proteins were transferred using a transfer buffer (160 mM glycine, 25 mM Tris, and 20% (v/v) methanol) at 90 V for 30 minutes using the Bio-Rad semiwet transfer system (Bio-Rad Laboratories USA Inc.). Membranes were stained with Ponceau-S solution (0.1% Ponceau red (Bio-Rad Laboratories USA Inc.) and 10% acetic acid (Labsynth Ltda.) and subsequently incubated for 16–18 hours at 4°C with a blocking solution (5% skimmed milk (Molico^®^, Nestlé S.A.), 2.5% (w/v) bovine serum albumin (Sigma-Aldrich) in PBS). Next, membranes were incubated for 1 hour at room temperature (rt) with an individual monoclonal anti-PvAMA-1 (domain II) antibody (K_2_43) at a dilution of 1:2,000 (v/v). After 3 washings of 10 minutes each with PBS-T solution (0.05% Tween 20 (v/v), Invitrogen, Life Technologies Corporation USA Inc.), the membrane was incubated with a peroxidase-conjugated anti-mouse IgG antibody (KPL, Kirkegaard & Perry Laboratories USA Inc.) diluted 1:3,000 in blocking solution for 1 hour at rt. After 3 washing steps with PBS-T solution, the membrane was rinsed with a developing solution from the SuperSignal kit (Thermo Fisher Scientific USA Inc.) until the bands appeared.

### Detection of Human IgG Specific to PvAMA1 Variants

Human IgG antibodies against PvAMA-1 variants were detected through ELISA as previously described (Múfalo et al., 2008). ELISA plates (High binding, Costar, EUA) were coated with 200 ng/well of each recombinant protein that was incubated overnight at rt. Next, plates were washed with PBS-T solution (0.05%-Tween 20^©^, Sigma-Aldrich) and blocked with PBS-milk solution (5% w/v, Molico^®^, Nestlé S.A.) for two hours at 37°C. Serum samples were diluted 1:100 in PBS-milk solution, and 50 μL of each sample was added to each well in duplicate. After incubation for one hour at rt followed by 5 washes with PBS-T, 50 μL of a solution containing peroxidase-conjugated goat anti-human IgG (Sigma-Aldrich), diluted 1:80,000, was added per well. The enzymatic reaction was developed by the addition of 1 mg/mL of o-phenylenediamine (Sigma-Aldrich) diluted in phosphate-citrate buffer, pH 5.0, containing 0.03% (v/v) hydrogen peroxide and was stopped by the addition of 50 μL of 4 N H_2_SO_4_. Plates were read at 492 nm (OD_492_) with an ELISA reader (Thermo Fisher Scientific USA Inc). The cutoff calculation was stipulated as three times the standard deviations (SD) above the mean values of the OD_492_ of 20 malaria-naive individuals from the state of São Paulo (Brazil). The cutoff values for each variant were as follows: Belem (0.125), Sal 1 (0.120), Chesson I (0.127), Indonesia XIX (0.091), SK0814 (0.133), TC103 (0.120), PNG_05_ESP (0.090), PNG_62_MU (0.082) and PNG_68_MAS (0.077). The reactivity index was calculated individually, with the OD value being divided by the cutoff value. After that screening step, serum samples were separated into three groups: i) lack of antibody reactivity, ii) low antibody reactivity (defined as below the OD_492_ < 0.8) and iii) high antibody reactivity (defined as above the OD_492_ > 0.8).

### Immunization of Mice and Cross-Reactivity Studies

Six- to eight-week-old female C57BL/6 mice were purchased from the animal facility of the School of Pharmaceutical Sciences/Chemistry Institute (University of Sao Paulo). Briefly, mice were subcutaneously (s.c.) immunized three times with an interval of 21 days for each dose of the vaccine formulation, which was composed of a recombinant PvAMA-1 protein and the Poly I:C adjuvant (*n* = 6 mice per group). For each dose, a final volume of 100 µL (10 µg of protein, 50 μg of Poly (I:C) HMW (Polyinosinic-polycytidylic acid - high molecular weight) (Invitrogen, Life Technologies Corporation USA Inc.) and sterile PBS) were s.c. injected into the left or right body flank, alternating them between each dose. Controls received only the adjuvant diluted in PBS. All animal experiments were approved by the Animal Care and Use Committee of the University of São Paulo (CEUA/FCF 32.2016-P520).

Anti-PvAMA-1 specific antibodies were detected through ELISA as previously described (Gimenez et al., 2017). Briefly, blood was collected from the submandibular vein of immunized mice 20 days after each vaccination, and the sera were obtained. Following an overnight incubation with 200 ng/well of each PvAMA-1 protein variant at rt, plates were washed with PBS-T solution and blocked with PBS-milk solution for 2 hours at 37°C. Pooled serum samples derived from each dose vaccination were analyzed for the presence of anti-PvAMA-1 antibodies. Serial dilutions of polyclonal sera beginning at 1:200 were added to the wells and incubated for 1 hour at rt. After a washing step with PBS-T solution was performed, peroxidase-labeled goat anti-mouse IgG (Sigma-Aldrich) solution was added to each well at a 1:3,000 dilution. Next, the OPD/acid stop system was utilized to determine the titers of anti-PvAMA-1 antibodies for each allelic variant based on the highest serum dilution that yielded an OD_492_ value higher than 0.1.

Inhibition assays were performed as previously described (Soares et al., 1999; Múfalo et al., 2008). Briefly, ELISA plates were coated with the recombinant PvAMA-1 Belem protein. The different PvAMA-1 protein variants (10 mg/mL) were previously incubated for one hour at rt with polyclonal sera mice immunized with PvAMA-1 Belem. This mixture (containing 1:100 serum dilution) was added to each well and incubated for one hour at rt. ELISA was subsequently performed as described above. The percentage of inhibition for each serum sample was calculated as follows: [OD_492_ in the presence of inhibitor/OD_492_ in the absence of inhibitor] x 100.

### Statistical Analysis

Statistical analysis was performed using Prism 8.3.0 (GraphPad Software). Fisher’s test was employed to compare the percentages of responders among groups. Comparisons of antibody levels were performed through the nonparametric Kruskal-Wallis test followed by Dunn’s multiple comparison test.

## Results

Nine PvAMA-1 sequences that were representative of the wide global distribution of *P. vivax*, including locations in Asia, Central and South America, and Oceania, were selected for this study (**Table 1**). Since the PvAMA-1 Belem variant was the only Brazilian representative from our protein portfolio, it was considered to be the reference protein in our analysis. Thus, we decided to initiate this study by determining the aa diversity in the ectodomain sequences of PvAMA-1 protein variants. In this approach, we also included the sequences of two recently described Brazilian isolates of *P. vivax* (PV11-101 (H5) and PV11-90 (H16)) (Bittencourt et al., 2020). A low level of PvAMA-1 polymorphism was found among the global isolates, ranging from 95.2% (Chesson I *versus* Indonesia XIX) to 99.6% (Belem *versus* TC103) (**Figure 1A**). To ensure that we could visualize the small differences among these PvAMA-1 protein sequences, we constructed a phylogenetic tree. Notably, PvAMA-1 isolates from different geographical locations shared the same cluster, such as i) Sal-1 and SK014; ii) PV11-101 (H5) and PNG_68_MAS. The remaining PNG isolates (PNG_05_ESP and PNG_62_MU) were observed to form a major cluster with Indonesia XIX and PV11-90 (H16) isolates. The Brazilian isolates (Belem, PV11-101 (H5) and PV11-90 (H16)) were all found in separated clusters in the phylogenetic tree (**Figure 1B**).

**Figure 2:**
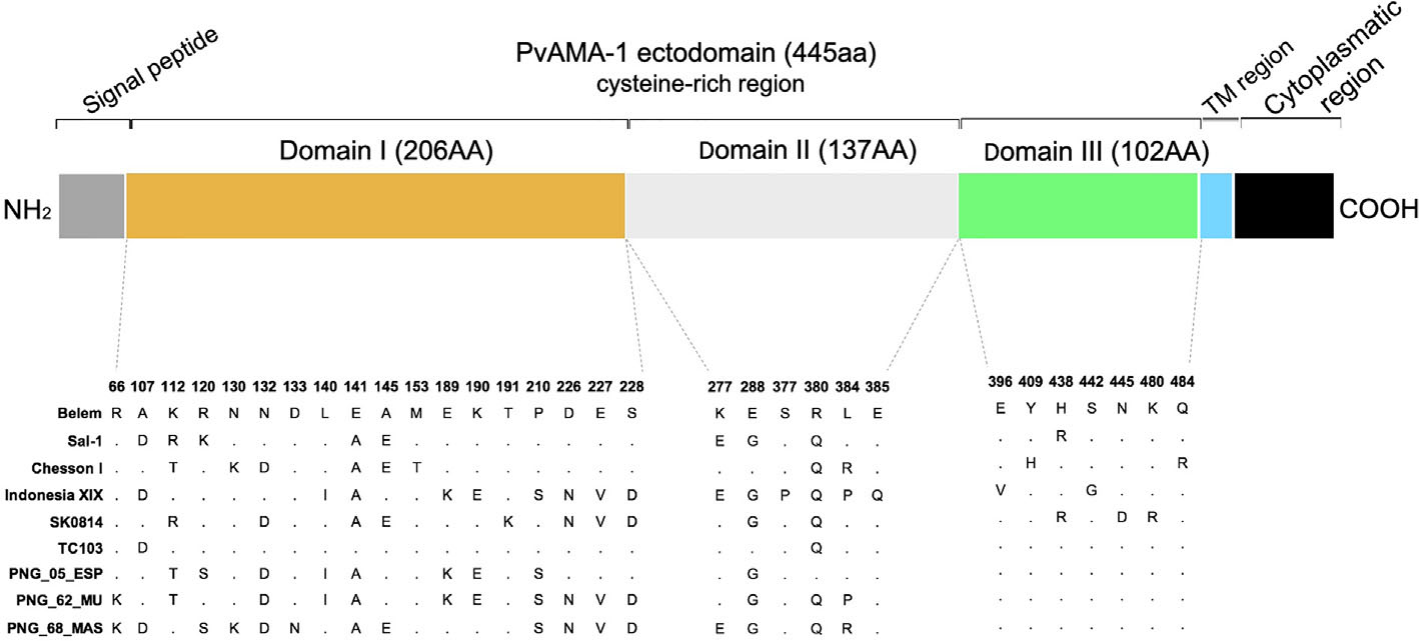
Schematic structure of PvAMA-1 and polymorphism display in comparison to the Belem variant. Signal peptide (gray), the ectodomain comprised domain I (yellow), domain II (light gray) and domain III (green), transmembrane region (blue) and cytoplasmic region (black). The polymorphic sites across the isolates are represented by amino acid letters, and the dots represent the nonpolymorphic sites.

In the past, our group expressed a PvAMA-1 protein (GenBank access number KJ010958, residues 43–487) utilizing the codon usage of *P. pastoris* without potential N-glycosylation sites (Vicentin et al., 2014). In this study, individual sequences from 9 PvAMA-1 representative alleles were cloned into the pPIC9K vector (Invitrogen) (**Table 1**) to obtain methanol-inducible expression in *P. pastoris* (all Mut+ phenotype). Recombinant proteins were successfully expressed at the ∼53 kDa molecular weight and migrating as single bands with barely detectable degradation products, and they were recognized by monoclonal anti-PvAMA-1 (domain II) antibodies (**Supplementary Figure 1**). Comparing the predicted aa sequences from the individual PvAMA-1 ectodomains (445 aa) of these 9 isolates, a total of 31 polymorphic sites were identified: 18, 7, and 6 in domains I, II, and III, respectively. These polymorphic residues were predominantly divalent, except for positions 112, 120 and 384, which were trivalent (**Figure 2**).

**Figure 3:**
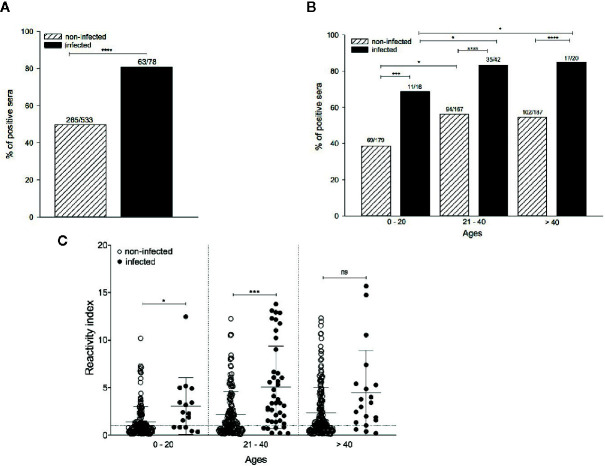
Comparison of the anti-PvAMA-1 Belem IgG response in infected and noninfected individuals naturally exposed to malaria. Serum samples from 611 individuals were grouped into i) infected at the moment of collection (n=78) and ii) noninfected at the moment of collection (n=533). The samples were analyzed for the presence of IgG antibodies against the protein PvAMA-1 Belem through ELISA. The diagnosis of infected individuals was performed using the thick smears technique in the act of collection. The cutoff value (0.125) was calculated using 20 sera from individuals from the city of São Paulo who had never been exposed to malaria. **(A)** Percentage of positive sera against the Belem variant. ****p < 0.0001. **(B)** Percentage of positive individuals whose antibodies recognized the Belem variant by age (0 - 20 years, 21 - 40 years or >40 years). *p < 0.05, ***p < 0.001, ****p < 0.0001. **(C)** Reactivity index (RI) in individuals against the Belem variant grouped by age. The dotted line represents RI equal to 1. The horizontal lack lines over the symbols indicate the median with a 95% confidence interval. ns, non significant; *p > *0.05, *p < 0.05, ***p < 0.001.

Next, we characterized the anti-PvAMA-1 IgG responses against the Belem strain in serum samples of 611 individuals from the state of Pará, Brazil (located in the Amazon region). Most of these individuals (328/611) presented anti-PvAMA1 binding IgG, corresponding to 53.68% of the entire cohort. Among infected and non-infected individuals, the percentage of positive sera for anti-PvAMA1 binding IgG was significantly higher during the patent infection (80.77%) than in non-infected counterparts (49.72%) (*p* < 0.0001 - **Figure 3A**), confirming the immunogenicity of the PvAMA-1 protein during natural infections (Rodrigues et al., 2005; Morais et al., 2006; Wickramarachchi et al., 2006; Múfalo et al., 2008; Vicentin et al., 2014; Xia et al., 2015; Rosas-Aguirre et al., 2015; Salavatifar et al., 2015; Sánchez-Arcila et al., 2015; Kale et al., 2019; Keffale et al., 2019; Surendra et al., 2019). The prevalence of these antibodies significantly increased with age for either infected or noninfected individuals, ranging from 68.8%/38.5% in children and youngsters (0-20 years old) to 83.3%/56.3% in young adults (21-40 years old), respectively (noninfected 0-20 years old vs noninfected 21-40 years old - *p* < 0.05; infected 0-20 years old vs infected 21-40 years old - *p* < 0.05; infected 0-20 years old vs infected > 40 years old - *p* < 0.05 - **Figure 3B**). Regarding infected and noninfected individuals > 40 years old who positively recognized PvAMA-1 Belem protein with serum IgG, their percentages did not differ from those of younger counterparts. Moreover, the prevalence of positive responders to the PvAMA-1 Belem protein was significantly higher in malaria-infected individuals than in noninfected individuals (0-20 years old, *p* < 0.001; 21-40 years old, *p* < 0.0001; > 40 years old, *p* < 0.0001; **Figure 3B**). Similar to the percentage of positive sera (**Figures 3A, B**), the reactivity index of serum samples from malaria-infected individuals was significantly higher than that of noninfected individuals at both 0-20 and 21-40 years of age (*p* < 0.05 and *p* < 0.001, respectively). In contrast, no difference was observed between the responder frequency (**Figure 3B**) and reactivity index (**Figure 3C**) between the age groups of 21-40 years and > 40 years for both infected and noninfected groups, indicating that most individuals may become responders based on age, reaching a plateau at an older age (> 40 years) (**Figures 3B, C**).

**Figure 4:**
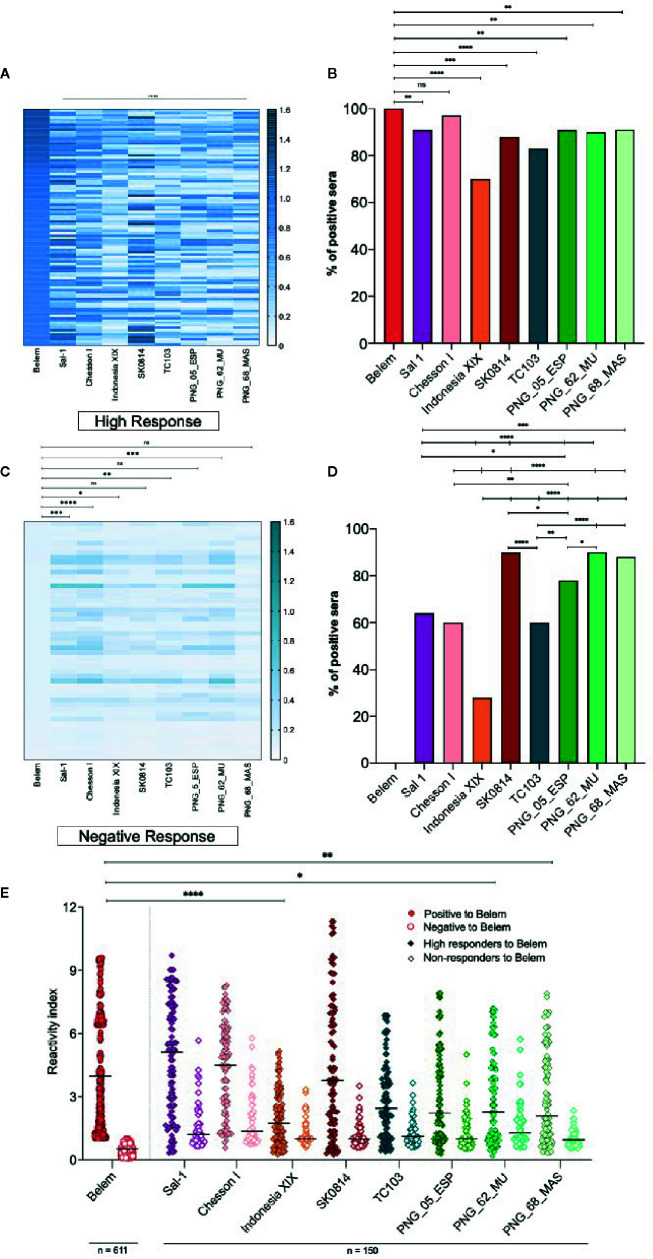
Comparison of the IgG antibody response against different PvAMA-1 variants in individuals exposed to malaria. **(A)** Heatmap of the anti-PvAMA-1 IgG magnitude derived from serum samples of 100 positive individuals for recognizing the PvAMA-1 Belem protein. These patients presented OD_492_ values >0.8 to PvAMA-1 Belem and were tested for the other variants: Sal 1, Chesson I, Indonesia XIX, SK0814, TC103, PNG_05_ESP, PNG_62_MU and PNG_68_MAS. Each line represents one sample, while the columns are representative of each protein variant. The closer the OD_492_ value is to 1.5, the darker the nuance in the graph. ****p < 0.0001. **(B)** Response against the variants studied in 100 individuals previously tested for the Belem strain. ns *p >*0.05, **p < 0.01, ***p < 0.001, ****p < 0.0001, Fisher´s test. **(C)** Heatmap of the IgG magnitude in samples from 50 serologically negative individuals for the PvAMA-1 Belem protein compared to other variants. Fifty patients with an OD_492_ value equal to or lower than the cutoff value (0.125) were tested for the other variants as described on item **(A)** ns, non significant, p>0.05, *p < 0.05, **p < 0.005, ***p < 0.001, ****p < 0.0001, Kruskal-Wallis. **(D)** Response against the variants studied in 50 individuals negative to the Belem strain. *p < 0.05, **p < 0.01, ***p < 0.001, ****p < 0.0001, Fisher´s test. **(E)** Reactivity index against the PvAMA-1 Belem protein (611 individuals) or against other variants (150 individuals: 100 positive to Belem strain and 50 negative to Belem strain). The horizontal black lines over the symbols indicate the median with a 95% confidence interval. *p < 0.05, **p < 0.005, ****p < 0.0001, Kruskal-Wallis test.

Considering all the PvAMA-1 ectodomain polymorphisms from the global isolates tested in this study (**Figure 2** and **Supplementary Figure 1**), we also investigated whether the serum samples of PvAMA-1 Belem variant responders and non-responders would bind to other variants. To this end, sera of 150 individuals (100 high responders that exhibited OD_492_ > 0.8 against the PvAMA-1 Belem protein through ELISA and 50 non-responders) were tested against different variants through ELISA. When ranked by the intensity of the antibody response against different variants, those 100 individuals with the highest anti-PvAMA-1 Belem IgG responses (OD_492_ values 0.8-1.2) exhibited a distinct profile among them. PvAMA-1 Sal-1, Chesson I and SK0814 were the variants recognized with the highest intensity (RI>3), while Indonesia XIX, PNG_62_MU and PNG_68_MAS variants were recognized by a considerably smaller number of these individuals (**Figures 4A, E**). Moreover, serum samples from these 100 high responders to the PvAMA-1 Belem variant presented similar positivity only against the Chesson I variant (**Figure 4B**). Thus, it is likely that PvAMA-1 Sal 1, Chesson I and SK0814 variants share common epitopes or epitopes of cross-reactivity with the Belem variant. Among the 50 residents from endemic malaria areas that tested negative for IgG anti-PvAMA-1 Belem variant, they recognized all remaining variants but with a very low OD for Indonesia XIX, TC103, and PNG_62_MU (**Figure 4C**). Despite the low reactivity (RI ≤ 3) (**Figure 4E**), more than 75% of these PvAMA-1 Belem non-responders showed positivity against SK0814 and all 3 PNG variants (**Figure 4D**). These data demonstrate that some PvAMA-1 polymorphisms have already been widely distributed geographically.

Next, we investigated whether this level of cross-reactivity against PvAMA-1 protein variants could also be seen after immunization. Pooled sera from C57BL/6 mice immunized thrice with the recombinant PvAMA-1 Belem adjuvanted with Poly (I:C) had their ability to recognize different PvAMA-1 variants detected by ELISA. As expected, the PvAMA-1 Belem variant was the major protein recognized by these samples followed by Chesson I, Indonesia XIX and SK0814 variants. In contrast, the PNG variants were only slightly recognized by pooled sera of immunized mice, except for PNG_05_ESP, which elicited an intermediate response. More specifically, the recognition of each PvAMA-1 protein variant was significantly different from that of Belem until the following serum dilutions: 1:800 to Indonesia (*p* < 0.0001); 1:1,600 to Sal-I (*p* < 0.0001), Chesson I (*p* = 0.0465), SK0814 (*p* = 0.0307) and TC103 (*p* < 0.0001); 1:6,400 to PNG_05_ESP (*p* < 0.009), PNG_62_MU (*p* < 0.0005) and PNG_68_MAS (*p* < 0.0003) (**Supplementary Figure 2**).

We also performed inhibition assays using PvAMA-1 Belem-coated plates as targets and different soluble PvAMA-1 protein variants as inhibitory molecules for polyclonal sera of PvAMA-1 Belem-immunized mice. Whereas the incubation of PvAMA-1 Belem protein with polyclonal serum almost completely inhibited the recognition of the PvAMA-1 Belem-coated plates (93.15% inhibition), other variants showed moderate to low inhibition of these plates (SK0814 - 72.8%, Indonesia XIX - 71.1%, Sal-1 - 54.87% and Chesson I 48.25%).

**Figure 5:**
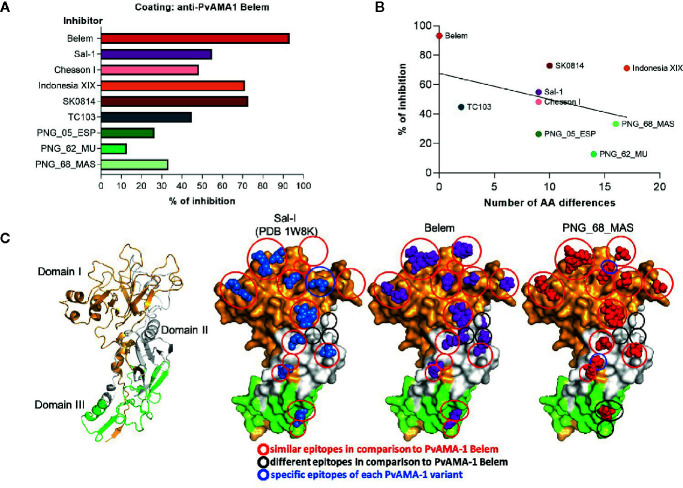
Influence of distinct PvAMA-1 polymorphisms on antibody recognition. **(A)** Inhibition percentage of PvAMA-1 Belem recognition by anti-PvAMA-1 Belem antibodies preincubated with other variants. **(B)** Correlation between the inhibition percentage of PvAMA-1 Belem recognition by anti-PvAMA-1 Belem antibodies preincubated with other variants and the number of different amino acids compared to the Belem variant (p-value = 0.28). **(C)** Tertiary protein structure models of three different Pv-AMA-1 isolates based on the crystal structure of the Sal-1 isolate (PDB ID: 1W8K). The domains are represented in gold (domain I), white (domain II) or green (domain III). The epitope residues are shown in blue (Sal-1), purple (Belem) or red (PNG_68_MAS). Red circles represent similar epitopes in comparison to Belem, black circles represent different epitopes and blue circles represent specific epitopes to each Pv-AMA-1 variant.

On the other hand, the incubation of polyclonal sera from PvAMA-1 Belem-immunized mice with TC103 or PNG isolates induced lower inhibition of PvAMA-1 Belem recognition, which ranged from 13 to 48% (**Figure 5A**). Of note, the higher the inhibition percentage of PvAMA-1 Belem recognition by polyclonal sera, the lower the number of distinct aa displayed by the other PvAMA-1 variant (**Figure 5B**). Therefore, these results indicated that the PvAMA-1 Belem variant apparently contains most of the epitopes present in the other allelic variants tested.

Since we evaluated the potential cross-reactivity between different epitopes of PvAMA-1 variants to extrapolate their effects under vaccination, we decided to predict the amino acids that would compose some of their respective conformational B cell epitopes. To this end, we employed the Discotope 2.0 server (http://www.cbs.dtu.dk/services/DiscoTope/) and the crystal structure of the PvAMA-1 ectodomain from the Sal-1 variant (PDB 1W8K). The analysis settings were performed with 80% specificity and 39% sensitivity (score < -2.5) and suggested a total of 33 residues with a high probability of being part of its epitopes. At the same time, we also employed the software ElliPro (http://tools.iedb.org/ellipro/) to predict discontinuous (conformational) epitopes with the same protein tridimensional structure. A total of 16 residues displayed the highest scores in the analysis. Considering all the residue positions as fixed for all PvAMA-1 variants, we identified their locations after aligning all sequences (**Supplementary Figure 3**). In this analysis, we also included the known PvAMA-1 epitope described for the F8.12.19 Fab (Igonet et al., 2007). Based on the identified epitope locations, we wanted to display their positions within the 3D protein structure of PvAMA-1. However, only the PvAMA-1 Sal-1 variant had crystallography data available (PDB 1W8K). As the design of tridimensional protein structures has been primarily performed with this type of data, we had to construct tertiary protein models for Belem and PNG_68_MAS. To this end, we utilized the iTasser server (Yang et al., 2014) and submitted these PvAMA-1 polymorphisms to different analyses with DisEMBL, WHATCHECK and QMEAN tools (Hooft et al., 1996; Benkert et al., 2011). The models of the tertiary structures were of robust quality and were sufficiently consistent to compare with the reference X-ray structures (PDB 1W81 and PDB 1W8K). Consecutive rounds of energy minimization were performed to correct angles and distance of the atom bonds and to evaluate the impact of mutated residues in the global structure. As expected, minor conformational changes were observed, since these residues are exposed to the solvent and mainly in flexible loops, especially in those that mediate protein-protein interactions (PPIs), regions known to be flexible. In relation to the Sal-1 variant sequence, the most notorious mutation in the PPI region of the Belem variant was the Asp replacement by an Ala in position 65. In PNG variants, several mutations could be annotated: Lys was replaced by a Ser in position 88, Asn-Asp by Asp-Asn in 90-91 and a Pro by a Ser in 168 (data not shown). We grouped the described epitopes into 3 categories: i) similar epitopes; ii) different epitopes (in relation to PvAMA-1 Belem); and iii) specific epitopes for each PvAMA-1 variant. Although the majority of epitopes seemed similar among the 3 variants, important differences in the epitope display for each variant could be detected in all 3 domains of the PvAMA-1 ectodomains (**Figure 5C**). Although the polymorphisms largely affected the non-epitope regions of PvAMA-1 (**Supplementary Figure 3**), they could have changed the epitope display. Whereas the Sal-1 variant had only a single changed epitope region, PNG_68_MAS presented 2 regions different than those of the Belem counterpart. Furthermore, both Sal-1 and PNG_68_MAS showed other regions without epitopes seen in the Belem variant. Although PvAMA-1 polymorphisms do not induce major conformational changes in protein folding, they indeed affect epitope display, which is critical for the development of a universal vaccine against *P. vivax* malaria.

## Discussion

A great part of the research attempting to develop a vaccine against *P. vivax* has been highly neglected, including that about the impact of PvAMA-1 allelic variants on the antibody acquisition. Overall, AMA-1 is a highly promising antigen to be included in a *P. vivax* malaria vaccine due its ability to produce high antibody titers, which can inhibit parasite invasion *in vitro* (Bouillet et al., 2011; Vicentin et al., 2014). This antigen has continually attracted several important research groups to the development of a malaria vaccine. These laboratories are employing different approaches to overcome issues imposed by PfAMA-1 allelic polymorphisms (Drew et al., 2012; Faber et al., 2013; Miura et al., 2013; Ouattara et al., 2013). More specifically, a recent study mapped several aa variations as the cause of strain-specific resistance caused by parasites isolated from individuals vaccinated with FMP2.1/AS02a (Malkin et al., 2008). These findings support the notion that select AMA-1 alleles must be included in vaccine formulation protection against this parasitic disease. Considering that PvAMA-1 sequences display limited polymorphisms (Gunasekera et al., 2007; Arnott et al., 2013; Bittencourt et al., 2020), the possibility emerges that few recombinant proteins may represent the key alleles needed in a vaccine to cover the entire population of *P. vivax*.

Our group previously showed that a recombinant protein encoding the ectodomain of a Brazilian PvAMA-1 isolate could be recognized by serum IgG antibodies in a large fraction of malaria-infected individuals (Rodrigues et al., 2005; Morais et al., 2006; Vicentin et al., 2014). We also reported that recombinant proteins based on the PvAMA-1 sequence could elicit high antibody titers when applied either as a homologous or heterologous regimen of vaccination in mice (Bouillet et al., 2011; Vicentin et al., 2014). These high IgG titers were obtained through vaccination and were associated with the usage of adjuvants licensed for human use: alum, MPLA, or the combination of alum plus MPLA (Vicentin et al., 2014; Thera et al., 2016).

Furthermore, the genetic diversity of PvAMA-1 does not seem to drastically interfere with the recognition of its respective variants. Serum samples of mice immunized with a protein based on an Amazonian *P. vivax* isolate recognized the native AMA-1 protein from Thai parasitic isolates (Vicentin et al., 2014; Rocha et al., 2017). Moreover, these antibodies could also inhibit the reticulocyte invasion of 4 different Thai isolates (Vicentin et al., 2014). Therefore, identifying key polymorphisms related to strain-specific immunity is crucial for designing a universal PvAMA-1 vaccine.

In the present study, we evaluated the immunogenicity degree of 8 PvAMA-1 variants from different regions of the globe relative to the Brazilian Belem strain that composes the vaccine formulation proposed by our group. A comparative sequence analysis showed that a higher degree of polymorphisms among these PvAMA-1 variants was found in domain I (18 sites) followed by domains III and II (7 and 6 polymorphic sites, respectively) (**Supplementary Figure 3**). Similar data were observed in the exposed domain I of PfAMA-1, which displayed a higher number of polymorphic residues than other domains (Guy et al., 2018). Despite the low variability of domain III residues among PvAMA-1 variants, it remains to be elucidated whether their epitopes play a role in protection, as described for PfAMA-1 through reverse immunodynamics (Spensley et al., 2019). Notably, some variants from closer locations were eventually clustered in distinct branches of a phylogenetic tree (**Figure 1B**), suggesting that they may have circulated in other regions before having their origin identified.

Regarding the humoral responses against the PvAMA-1 Belem variant, our analyses demonstrated that host age has a direct influence on the magnitude of the response and the percentage of responders among infected patients from the Amazon region (**Figures 3B, C**). The 53.68% of positive individuals who recognized the PvAMA-1 Belem variant in this study represent a slightly lower percentage than those registered several years ago in the same region, which ranged from 59% to 70% (Morais et al., 2006; Múfalo et al., 2008; Vicentin et al., 2014). Corroborating these data, microarray analysis demonstrated that sera of Malian adults displayed higher magnitude and breadth of reactivity against peptides representing different PfAMA-1 polymorphisms than did children. Similar patterns of response were observed with samples collected during the peak season relative to preseason (Bailey et al., 2020). Since multiple parasite exposure tends to occur by the years in malaria endemic areas, increasing antibody titers and ensuing immunity in adults above 40 years of age, these data were expected (Silveira et al., 2018). Higher anti-PvAMA-1 antibody titers have already been positively correlated with an enhanced number of malaria episodes (Rodrigues et al., 2005).

After we defined the positive responders to the Belem variant in our cohort, we determined how these individuals would respond to other PvAMA-1 variants. Sal-1, Chesson I and SK0814 showed the highest correlation and reactivity index to that found for Belem recognition, indicating that they might share common epitopes. In contrast, more than 75% of the non-responders to Belem had their sera recognizing SK0814, and all tested PNG variants had a much lower reactivity index (**Figures 3B, C**). After narrowing the pattern of humoral response detected against PvAMA-1 variants in the context of human infection, we detected a similar response pattern under mouse immunization. Serum antibodies of animals immunized with the recombinant PvAMA-1 Belem protein and Poly (I:C) showed binding antibodies to Chesson I and SK0814 proteins. Disregarding the blood sample analyzed (human or mouse), we found that at least Chesson I and SK0814 PvAMA-1 proteins were highly recognized, reinforcing the hypothesis that these variants share common epitopes with the Belem counterpart. In contrast, these serum samples poorly recognized the PNG variants, which also displayed the lowest inhibition rates in competition ELISA assays (**Figure 5A**). In fact, the inhibition percentage related to Belem recognition tends to be inversely correlated with the number of polymorphic aa present in the tested variants (**Figure 5B**).

A recent study identified polymorphisms predominantly in regions of B cell epitopes of PvAMA-1, using only Brazilian isolates (Bittencourt et al., 2020). Thus, we decided to investigate this scenario by predicting the location of conformational B-cell epitopes in tridimensional structures of Belem as well as in foreign variants with similar and distinct humoral responses (Sal-I and PNG_68_MAS, respectively). Most epitopes found in these 2 variants were similar to those displayed by the Belem variant. However, Sal-1 still displayed a total of 9 distinct aa (**Figure 5B**) and an intermediate ability to inhibit the recognition of Belem protein by antibodies (**Figure 5A**). Additionally, this variant presented 1 specific epitope located in domain I and lacked 3 epitopes from domain II in comparison to the Belem epitopes (**Figure 5C**). Although the PNG_68_MAS variant had more than 15 aa with sequence differences (**Figure 5B**), the variant showed 2 specific epitopes in domains I and II, 1 different epitope from domain III, and lacked 4 epitopes in domains II and III in comparison to the Belem epitopes (**Figure 5C**). Thus, our study demonstrated that most PvAMA-1 polymorphisms affected non-epitope regions (**Supplementary Figure 3**).

Taken together, these data suggest that B cell epitopes are widespread among the global PvAMA-1 variants analyzed. Whereas mutations at mapped epitopes may influence the antibody recognition, if they occur in non-epitope sites, they may alter the conformational target structure and epitope display. As consequence, this phenomenon may lead to impairment of antibody recognition. Hence, we conclude that combination of PvAMA-1 variants should be further investigated as a multiallelic vaccine formulation to increase its chances of covering the needed breadth of the response to provide protection against this globally distributed pathogen.

## Data Availability Statement

The datasets presented in this study can be found in online repositories. The names of the repository/repositories and accession number(s) can be found in **Table 1**.

## Ethics Statement

The studies involving human participants were reviewed and approved by the Ethics Committee of the Evandro Chagas Institute (020/2006 and 0031/2010) and the School of Pharmaceutical Sciences, University of São Paulo (CAAE no. 3.198.871/2019). The patients/participants provided their written informed consent to participate in this study. The animal study was reviewed and approved by the Animal Care and Use Committee of the University of São Paulo (CEUA/FCF 32.2016-P520).

## Author Contributions

MGC and ISS designed the study and associated protocols. ACBF, KSF and RFM performed research work. ACBF, KSF, GHGT, RAG, ELVS, and ISS analyzed the data. MMP and MGC contributed the reagents and materials. KSF, ELVS, and ISS wrote the manuscript. All authors contributed to the article and approved the submitted version.

## Funding

This work was supported by grants from Fundação de Amparo à Pesquisa do Estado de São Paulo (FAPESP 2012/13032-5 and 2016/50108-0), Instituto Nacional de Ciência e Tecnologia de Vacinas (INCTV), National Counsel of Technological and Scientific Development (CNPq – 555.654/2009-5), and Pará State Research Foundation (FAPESPA – ICAAF 005/2011). ACBF, RAG and ISS are recipients of fellowship from CNPq. RFM is recipient of a fellowship from Coordenação de Aperfeiçoamento de Pessoal de Ensino Superior (Capes).

## Conflict of Interest

The authors declare that the research was conducted in the absence of any commercial or financial relationships that could be construed as a potential conflict of interest.
