# Supplementary figures and images for: Antibodies Against the Plasmodium vivax Apical Membrane Antigen 1 From the Belem Strain Share Common Epitopes Among Other Worldwide Variants
Source: Front Cell Infect Microbiol. 2021 Mar 16;11:616230. doi: 10.3389/fcimb.2021.616230 (PMC8009186; doi:10.3389/fcimb.2021.616230)

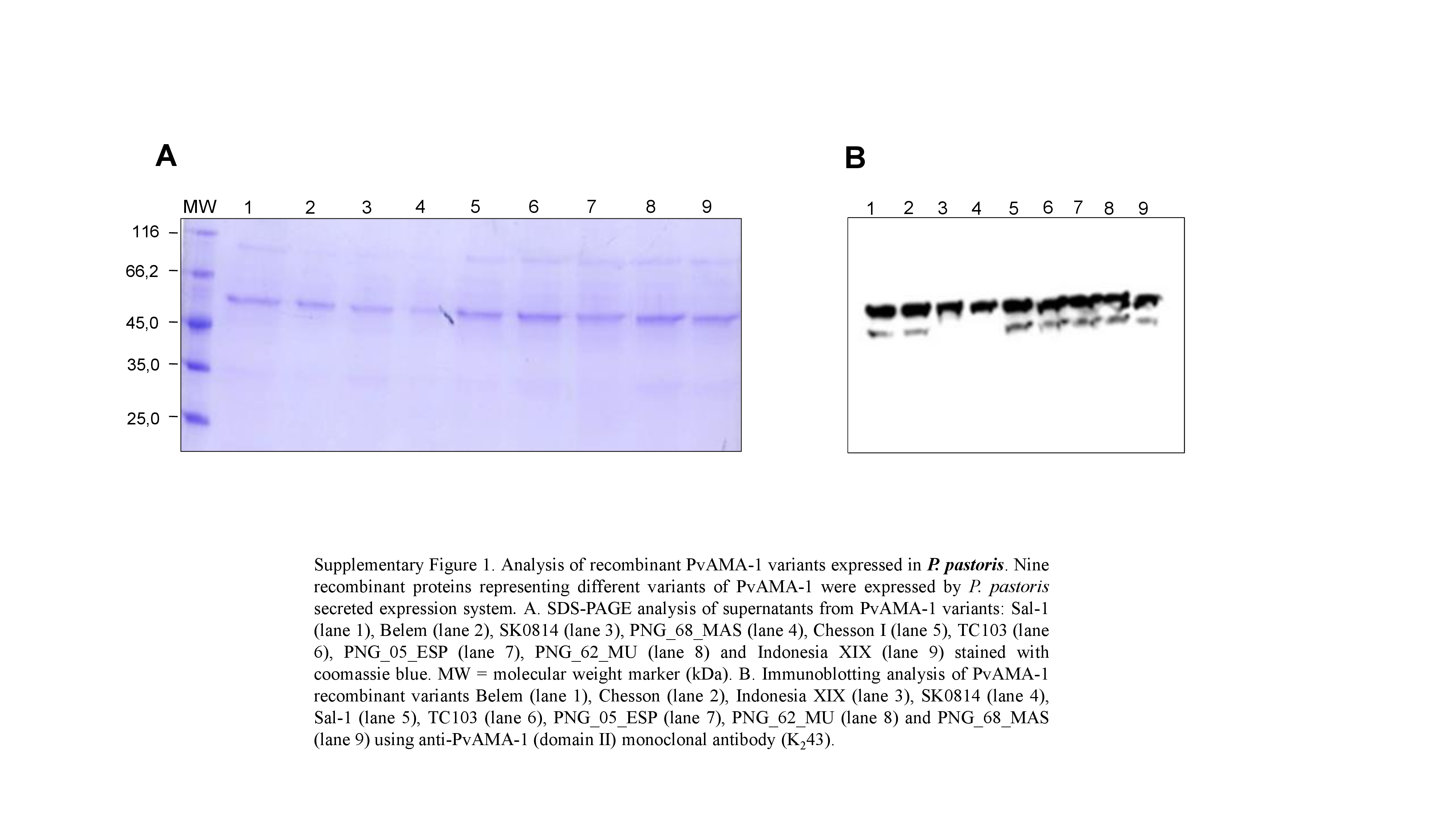

Supplement: Supplementary file 1 [file Image_1.tiff]

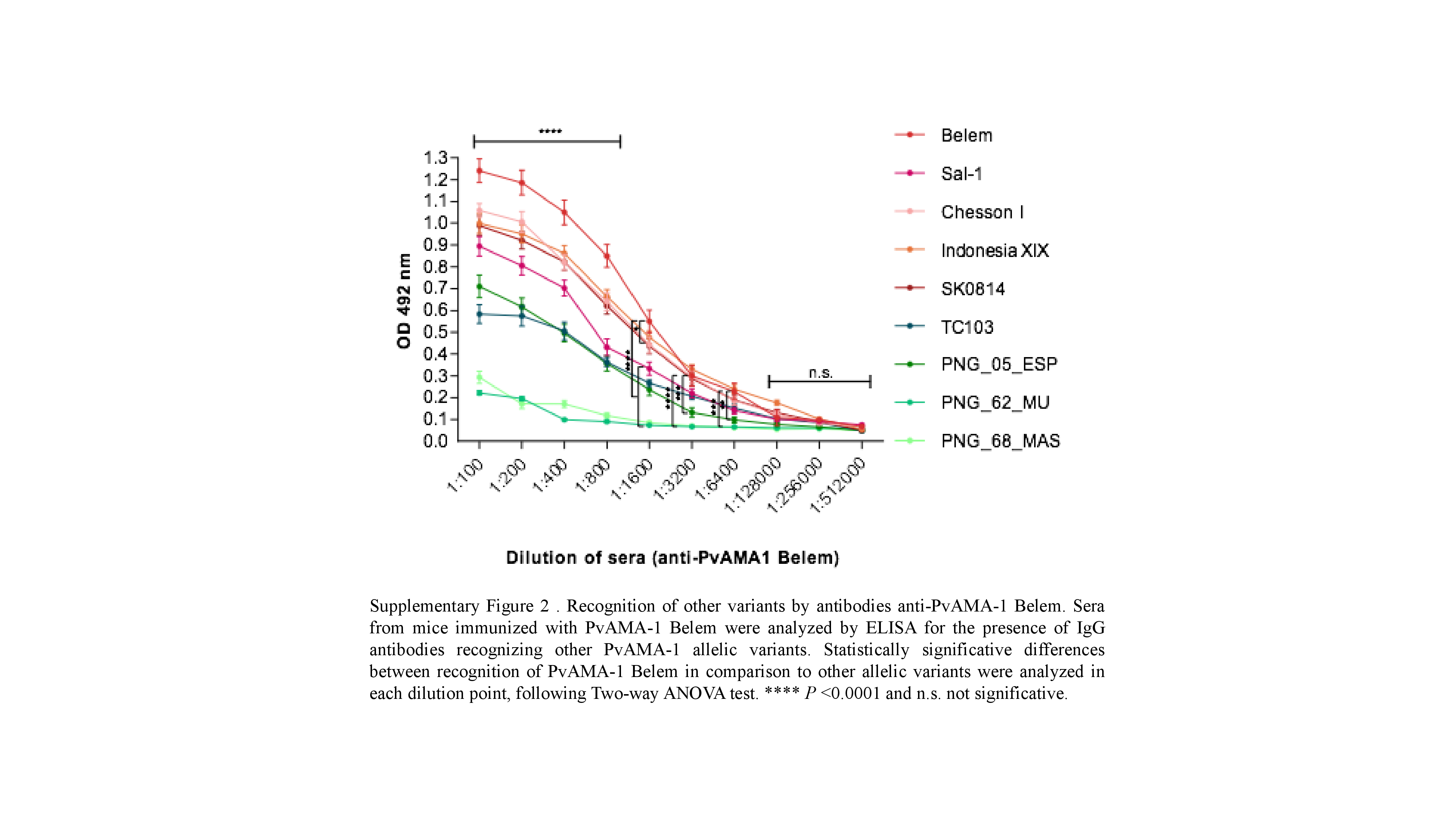

Supplement: Supplementary file 2 [file Image_2.tiff]

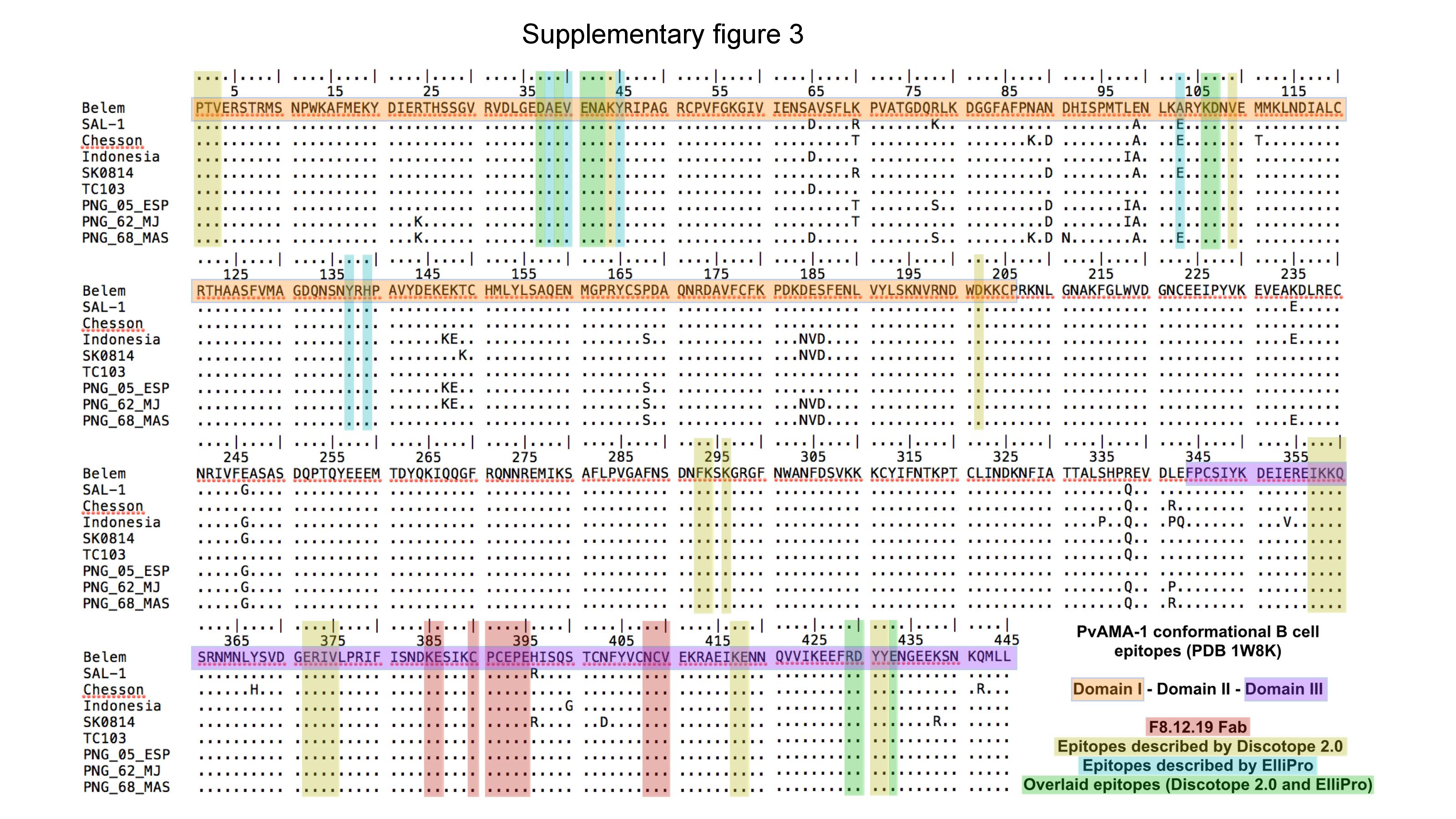

Supplement: Supplementary file 3 [file Image_3.tiff]
